# Robot versus video-assisted thoracoscopic thymectomy for large thymic epithelial tumors: a propensity-matched analysis

**DOI:** 10.1186/s12893-023-02228-8

**Published:** 2023-10-27

**Authors:** Long-fei Zhu, Ling-min Zhang, Chun-jian Zuo, Tian-yu Sun, Bin Jiang

**Affiliations:** Department of Thoracic Surgery, Daping Hospital, Army Medical University, No. 10, Changjiang Route, Yuzhong District, Chongqing, 400042 China

**Keywords:** Thymic epithelial tumors, Thymectomy, Robot-assisted thoracoscopic Surgery, Video-assisted thoracoscopic Surgery, Perioperative outcomes, Propensity score match

## Abstract

**Background:**

Both video-assisted thoracoscopic surgery (VATS) thymectomy and robot-assisted thoracoscopic surgery (RATS) thymectomy have been suggested as technically sound approaches for early-stage thymic epithelial tumors. However, the choice of VATS or RATS thymectomy for large and advanced thymic epithelial tumors remains controversial. In this study, the perioperative outcomes of VATS and RATS thymectomy were compared in patients with large thymic epithelial tumors (size ≥5.0 cm).

**Methods:**

A total of 113 patients with large thymic epithelial tumors who underwent minimally invasive surgery were included. Sixty-three patients underwent RATS, and 50 patients underwent VATS. Patient characteristics and perioperative variables were compared.

**Results:**

Compared with the VATS group, the RATS group experienced a shorter operation time (median: 110 min vs.130 min; *P* < 0.001) and less blood loss (30.00 ml vs. 100.00 ml, *P* < 0.001). No patients in the RATS group needed conversion to open surgery, but in the VATS series, five patients required conversion to open procedures (0% vs. 14.29%, *P* = 0.054). The rate of concomitant resection in the RATS group was similar to that in the VATS group (11.43% vs. 5.71%; *P* = 0.673). There was no significant difference between the two groups in the duration of chest tube (*P* = 0.587), postoperative complications (*P* = 1.000), and the duration of postoperative hospital stay (*P* = 0.141).

**Conclusion:**

For large thymic epithelial tumors, RATS thymectomy can be performed safely and effectively in a radical fashion. Due to the advanced optics and precise instrument control, concomitant resections can be easily achieved in larger thymic epithelial tumors using the robotic approach.

## Background

Despite being a relatively rare neoplasm, thymoma which originates from the epithelial cells of the thymus is the most common tumor of the anterior mediastinum in the adult population [1, 2]. Currently, the treatment of thymoma and thymic carcinoma mainly involves surgical resection, chemoradiotherapy, immunotherapy, and tyrosine kinase inhibitors. In terms of resectable tumors, thymectomy which is traditionally performed through a median sternotomy is the primary treatment [3, 4]. With the evolution of surgical technology and instruments, video-assisted thoracoscopic surgery (VATS) thymectomy is becoming the preferred surgical approach for early-stage thymic epithelial tumors because of less intraoperative bleeding and accelerated recovery compared to the classical transsternal approach [5]. However, VATS is also limited by its 2-dimensional view and the feasibility of complete dissection, especially for advanced thymic epithelial tumors. Robot-assisted surgical systems provide surgeons with 3D-magnified imaging, increased precision and instrument motion dexterity. Due to the potential great advantages, particularly in patients with large thymic epithelial tumors who require more precise dissection in a narrow anterior mediastinal space, robot–assisted thoracoscopic surgery (RATS) thymectomy has gradually emerged as an alternative approach over the last few years [6].

In existing articles, some investigators have shown that both RATS and VATS thymectomy appear feasible and safe for the resection of early-stage thymic epithelial tumors with comparable surgical outcomes [7, 8]. However, the literature pertaining to complete resection of large thymic epithelial tumors (maximal diameter ≥5.0 cm) through VATS or RATS is very limited, and few studies have compared the perioperative outcomes achieved with RATS to those achieved with VATS in patients with large tumors.

In the current retrospective study, we evaluated the safety and feasibility of RATS thymectomy in comparison with VATS thymectomy to explore the more appropriate approach for the treatment of large thymic epithelial tumors.

## Methods

### Patient collection

**Fig. 1:**
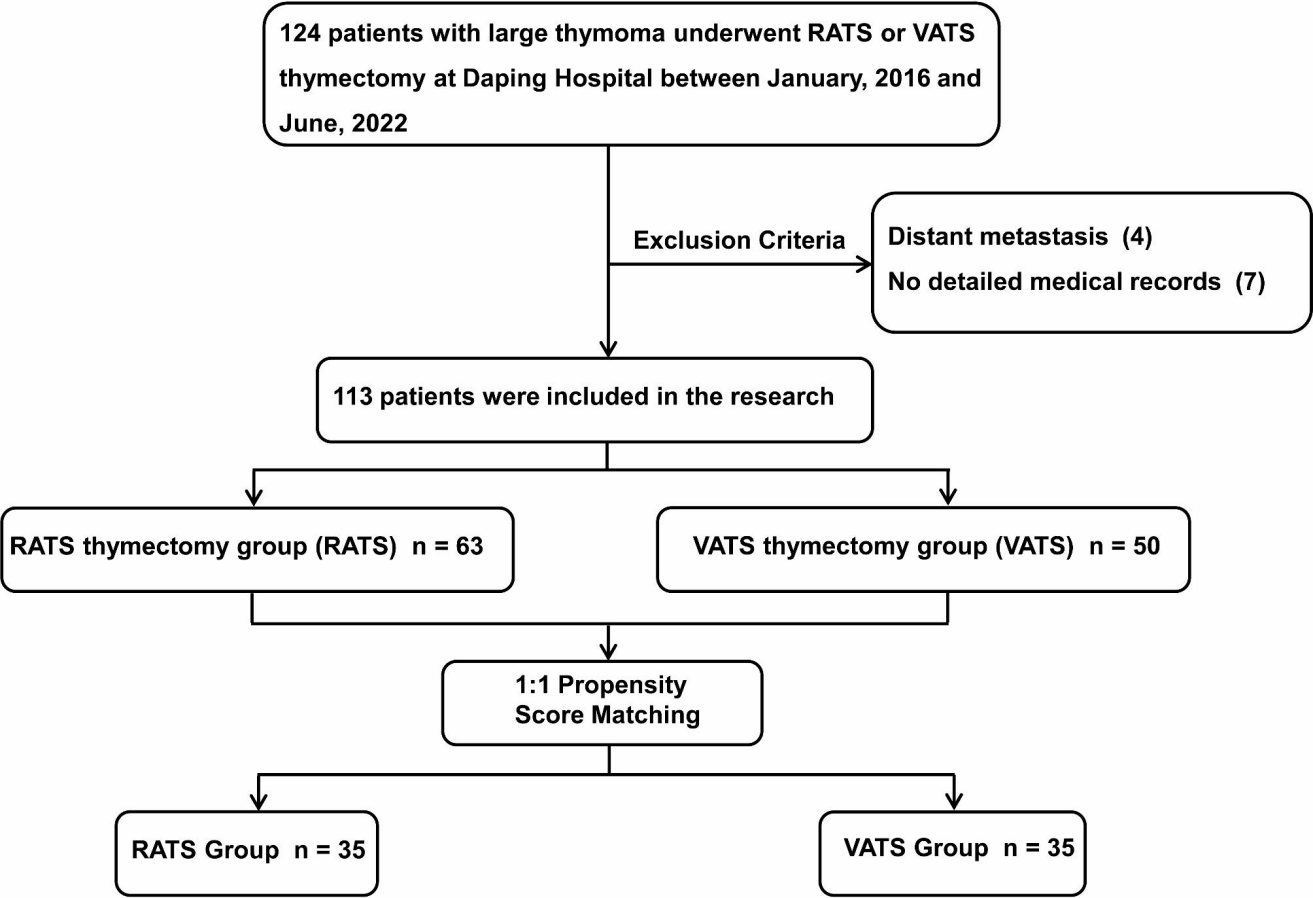
Flowchart demonstrating patient inclusion and exclusion. RATS, Robot-assisted thoracoscopic surgery; VATS, Video-assisted thoracoscopic surgery

All the patients included in this research fit the following criteria: (1) pathologically confirmed thymoma or thymic carcinoma with maximal diameter ≥5.0 cm; (2) treated with VATS or RATS thymectomy, including conversion during mini-invasive surgery; and (3) complete medical records. Distant metastasis detected prior to surgery was considered the exclusion criterion. Finally, 113 consecutive patients undergoing VATS or RATS thymectomy at Daping Hospital between January 2016 and June 2022 were included (Fig. 1). Whether the VATS or RATS thymectomy was performed was mainly determined by patients’ decisions after being informed the advantages and disadvantages of these two approaches. Patients were grouped according to the surgical approach, RATS and VATS groups. The operations in this cohort were performed by four different surgeons with a high level of homogeneity, each of them has more than 20 years clinical experience. All the patients signed a written consent for RATS or VATS surgery. This study was approved by the Ethics Committee of Daping Hospital (Ratification No. 2023(101)). The need for patient consent was waived because of the retrospective nature of the study.

All patients underwent enhanced chest computed tomography (CT) to evaluate the tumor size, location and potential invasion of adjacent structures. Cardiac and pulmonary functions and the symptoms of myasthenia gravis (MG) were fully evaluated before the operation, and the results indicated that all patients were eligible for surgery. Patients included in the study were staged according to the Masaoka staging system and histologic characteristics were confirmed based on the World Health Organization (WHO) classification.

### Surgical procedures

For transthoracic operations were performed under general anesthesia with double-lumen intubation, whereas subxiphoid approach was performed with single-lumen intubation. Asymptomatic thymomas were treated with thymectomy, aiming to completely remove the thymus and thymoma, while thymomas complicated with MG received extended thymectomy, including resection of the whole thymus, thymoma, anterior mediastinal fat and invaded adjacent structures. The principle of tumor-free manipulation was followed during all surgeries. Insufflation with carbon dioxide ranging from 8 to 10 mmHg was performed to create an optimal operative view and more operating space during the surgical procedures.

**Fig. 2:**
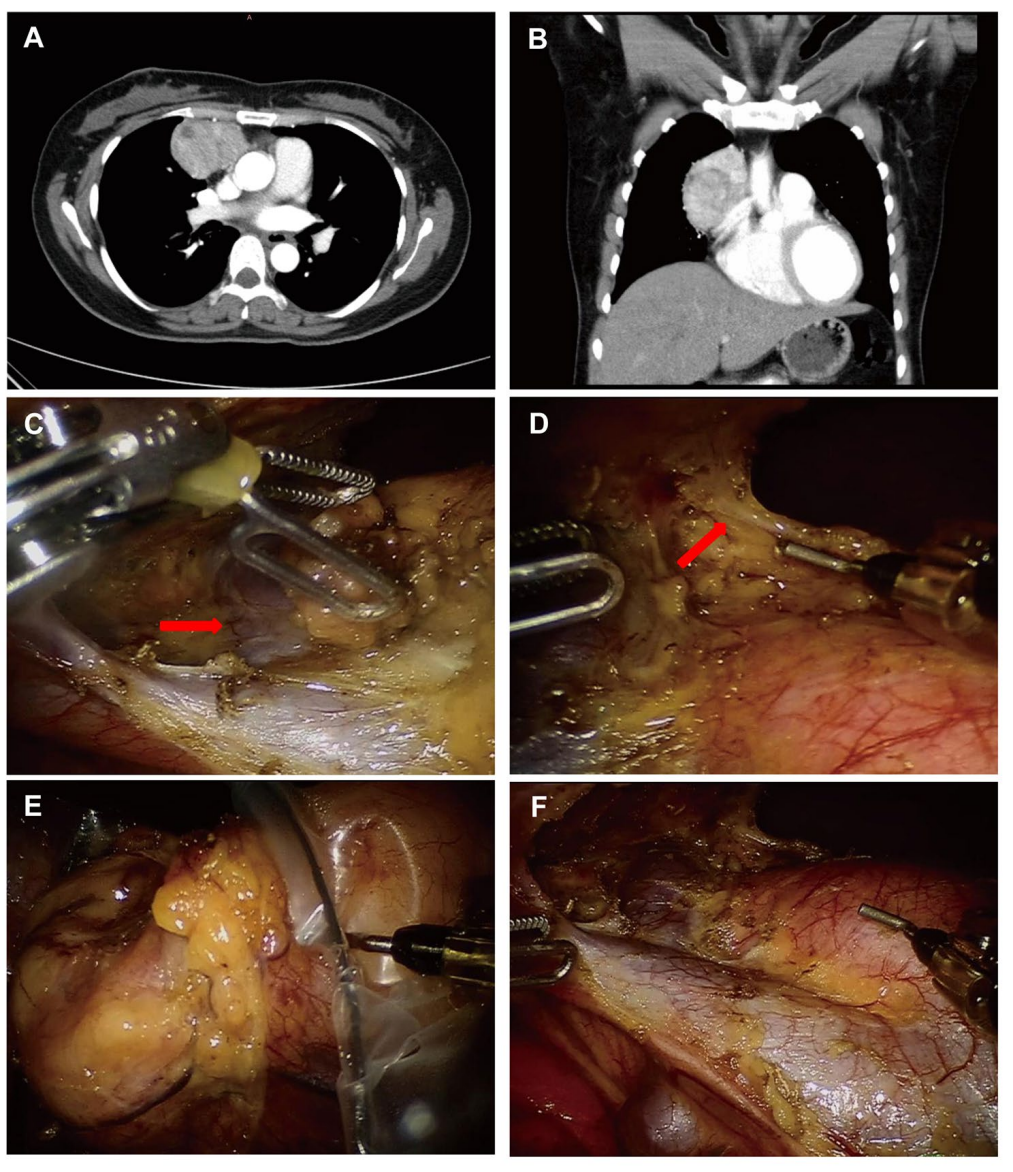
Robot-assisted thymectomy via right-side thoracic approach for a patient with large thymoma. (A) and (B) Preoperative computed tomographic scan of a 37-year old woman with a suspected thymoma (tumor size 4.1 × 5.3 × 6.5 cm). (C) The left innominate vein (red arrow) and superior vena cava were exposed clearly. (D) Intraoperative right thoracoscopic view of the left phrenic nerve (red arrow). (E) The thymoma was removed using a retrieval bag. (F) Surgical field after complete resection of the tumor

The Da Vinci Si system (Intuitive Surgical, Inc., USA) was used to perform the robot-assisted thymectomies. For the transthoracic approach, patients were positioned in the 30° lateral position with a cushion placed under the torso. The RATS were approached from either the left or right chest based on the predominant laterality of the tumor through three-port method with one assistant port if necessary. The robotic endoscope was inserted in the mid-axillary line in the 5th intercostal space, and two 8 mm working ports were placed at the 3rd and 4th intercostal spaces at the anterior axillary and mid-clavicular lines respectively. The assistant port was usually placed in the fourth intercostal space at the mid-axillary line. Most dissections during the RATS were performed with electrocautery and bipolar forceps (Fig. 2). For subxiphoid approach, patients were supine on the operating table with the dorsal elevated. A 3-port approach was commonly used with one potential assistant-port if necessary. A 1.5 cm vertical incision was made below the xiphoid to set the robotic endoscope. After dividing the rectus abdominis muscle, the retrosternal space was created by blunt finger dissection as previously described [9, 10]. Under the guidance of the finger, two additional robotic ports were placed below the bilateral costal arches at the mid-clavicular line. The potential assistant-port was usually placed in the 4th intercostal space at the mid-axillary line. Bilateral mediastinal pleura were opened during the operation, and the lower part of the sternum was slightly elevated by the camera arm as needed to widen the manipulation space. Electrocautery and bipolar forceps (Maryland) were also used.

The selection of surgical approach (transthoracic or subxiphoid approach) was based on the extent of tumor invasion of surrounding organs according to preoperative imaging examination, and the surgeons’ preferences.

For VATS thymectomy, the position during surgery, incision design and dissection were generally similar to those mentioned above in RATS. Notably, instead of bipolar forceps, ultrasonic dissector and electrocautery were used for resection.

For both VATS and RATS thymectomy, concomitant resection could be performed using endoscopic staplers when tumors invaded the lung, innominate vein, and even the superior vena cava. All specimens, including the thymus, thymoma and the surrounding adipose tissue, were removed using a retrieval bag through the assistant port to minimize the risk of the tumor cell spread and seeding. For larger tumors, the incision was enlarged avoiding excessive squeezing during retrieval processes. After adequate hemostasis, one drainage tube (24Fr) was inserted into the mediastinum in most patients, especially in patients who underwent concomitant resection. Chest tubes were not placed after surgeries in some patients from both groups based on the enhanced recovery after surgery (ERAS) protocol [11].

### Postoperative management

Chest radiography was performed for the evaluation on the same day as surgery. Nonsteroidal anti-inflammatory drugs (NSAIDs), mainly flurbiprofen axetil and parecoxib, were commonly given after surgery to relieve perioperative pain and reduce the stress response. Postoperative complications, including pulmonary inflammation, pneumothorax, hemothorax, chylothorax and myasthenia crises, were treated promptly and recorded in detail. The chest tube was removed from all patients when no air leak was observed and the production of pleural fluid was less than 200 mL per day.

### Statistical analysis

All statistical tests were performed using SPSS software, version 22.0 (IBM, Chicago, IL, USA). The continuous data are described as medians and interquartile ranges (IQRs) or means with standard deviations (SD). The Wilcoxon rank-sum test or LSD test (homogeneity of variance) was performed for comparisons between the two groups. Categorical variables are reported as counts and percentages of patients and were compared with the χ^2^ or Fisher’s exact test. The heterogeneity in baseline characteristics between the two groups (RATS group vs. VATS group) was balanced by performing 1:1 propensity score matching with a caliper width of 0.05. Propensity scores were based on age, sex, the tumor size, WHO classification, and Masaoka stage. *P* < 0.05 was considered statistically significant.

## Results

### Patient characteristics

The preoperative characteristics of the two groups of patients are shown in Table 1. Among the included patients, 63 underwent RATS, and 50 underwent VATS. The comorbidities of these patients mainly included hypertension, diabetes, coronary heart disease, chronic obstructive pulmonary disease and other conditions. The RATS group consisted of 44 men (69.84%), with a mean age of 49.48 ± 12.80 (range: 22 to 73) years, whereas the VATS group included more female patients (n = 29, 58.00%, *P* = 0.003), and the mean age was 51.42 ± 10.48 (range: 27 to 74, *P* = 0.388) years. Patients receiving RATS had a relatively larger tumor size than those in the VATS group (median 7.0 vs. 6.0 cm, *P* = 0.012). The distribution of the Masaoka stage between the two groups differed significantly (*P* = 0.048). No statistically significant differences in body mass index, underlying diseases, anesthesiologists physical status classification (ASA class), the presence of MG, and histological classification were observed between the two groups. Furthermore, two patients (3.17%) in the RATS group and one patient (2.00%, *P* = 1.000) in the VATS group received preoperative radiotherapy.

**Table 1:** Demographic characters of patients in the RATS and VATS group before and after 1:1 propensity score matching

Characteristics	Before propensity score matching			After propensity score matching		
	RATS group (n = 63)	VATS group (n = 50)	*P* Value	RATS group (n = 35)	VATS group (n = 35)	*P* Value
Age (years)	49.48 ± 12.80	51.42 ± 10.48	0.388	50.97 ± 11.98	49.20 ± 9.62	0.497
Gender			0.003			1.000
Male	44 (69.84)	21 (42.00)		18 (51.43)	18 (51.43)	
Female	19 (30.16)	29 (58.00)		17 (48.57)	17 (48.57)	
BMI (Kg/m^2^)	23.43 ± 2.94	23.84 ± 3.05	0.474	23.23 ± 2.94	24.11 ± 3.22	0.238
Comorbidity (%)			0.943			0.962
Hypertension	7 (11.11)	8 (16.00)		6 (17.14)	4 (11.43)	
Diabetes	2 (3.17)	2 (4.00)		2 (5.71)	1 (2.86)	
Coronary heart disease	3 (4.76)	1 (2.00)		0	0	
COPD	1 (1.59)	1 (2.00)		1 (2.86)	1 (2.86)	
Others	4 (6.35)	4 (8.00)		3 (8.57)	3 (8.57)	
ASA status class			0.455			1.000
I	32 (50.79)	29 (58.00)		19 (54.29)	20 (57.14)	
II	31 (49.21)	21 (42.00)		16 (45.71)	15 (42.86)	
III	0	0		0	0	
Tumor size (cm), median (IQR)	7.0 (6.0, 9.0)	6.0 (5.5, 7.0)	0.012	7.0 (6.0, 7.5)	6.5 (5.6, 7.6)	0.746
Myasthenia Gravis, n (%)	13 (20.63)	8 (16.00)	0.529	4 (11.43)	7 (20.00)	0.513
WHO Classification, n (%)			0.235			0.428
A	10 (15.87)	5 (10.00)		7 (20.00)	3 (8.57)	
AB	17 (26.98)	20 (40.00)		10 (28.57)	15(42.86)	
B1	7 (11.11)	11 (22.00)		6 (17.14)	7 (20.00)	
B2	20 (31.75)	11 (22.00)		11 (31.43)	7 (20.00)	
B3	5 (7.94)	2 (4.00)		1 (2.86)	2 (5.71)	
Thymic carcinoma	4 (6.35)	1 (2.00)		0 (0)	1 (2.86)	
Masaoka Stage, n (%)			0.048			0.439
I	36 (57.14)	40 (80.00)		25 (71.43)	28 (80.00)	
IIa	17 (26.98)	6 (12.00)		9 (25.71)	5 (14.29)	
IIb	4 (6.35)	3 (6.00)		1 (2.86)	2 (5.71)	
III	6 (9.53)	1 (2.00)		0 (0)	0 (0)	
IV	0 (0)	0 (0)		0 (0)	0 (0)	
Pre-operative treatment, n (%)			1.000			1.000
Yes	2 (3.17)	1 (2.00)		1 (2.86)	1 (2.86)	
No	61 (96.83)	49 (98.00)		34 (97.14)	34 (97.14)	

Abbreviations: BMI Body mass index, COPD Chronic obstructive pulmonary disease, ASA American Society of Anesthesiology, IQR interquartile range, RATS Robot-assisted thoracoscopic surgery, VATS Video-assisted thoracoscopic surgery

After PSM, the cohorts were narrowed to 35 patients in each group. All the baseline data, such as sex (*P* = 1.000), tumor size (*P* = 0.746), and the Masaoka stage (*P* = 0.439), were comparable between the two groups (Table 1). All further statistical analyses were performed on this population.

### Comparisons of intra- and postoperative parameters

When analyzing the operative outcomes in the propensity score-adjusted cohorts, we observed a statistically significant reduction in the operative time in RATS group (median: RATS, 110 vs. VATS, 130 min; *P* < 0.001). As shown in Table 2, the median operative blood loss during the RATS was 30.00 mL, compared to 100.00 mL during the VATS (*P* < 0.001). The mini-invasive laterality (*P* = 1.000) and complete resection rates (100.00% vs. 100.00%; *P* = 1.000) were statistically similar between the RATS and VATS groups.

**Table 2:** Operative and post-operative outcomes of the matched RATS and VATS groups

Variables	RATS group	VATS group	*P*-value
	(n = 35)	(n = 35)	
Mini-invasive laterality, n (%)			1.000
Right side	18 (51.43)	17 (48.57)	
Left side	13 (37.14)	13 (37.14)	
Sub-xyphoid	4 (11.43)	5 (14.29)	
Operative time (min), median (IQR)	110.00 (80.00, 125.00)	130.00 (100.00, 170.00)	< 0.001
Intraoperative blood loss (ml), median (IQR)	30.00 (20.00, 50.00)	100.00 (30.00, 200.00)	< 0.001
Conversion, n (%)	0 (0)	5 (14.29)	0.054
R0 resection, n (%)	35 (100.00)	35 (100.00)	1.000
Concomitant resection, n (%)	4 (11.43)	2 (5.71)	0.673
Lung	2 (5.71)	2 (5.71)	
Pericardium	3 (8.57)	1 (2.86)	
Innominate vein	2 (5.71)	0 (0)	
Phrenic nerve	1 (2.86)	1 (2.86)	
Without chest tube, n (%)	1 (2.86)	3 (8.57)	0.614
Duration of chest tube (days), median (IQR)	3.00 (2.00, 4.00)	3.00 (2.00, 4.00)	0.587
Total drainage volume (ml), median (IQR)	575.00 (400.00, 800.00)	400.00 (240.00, 630.00)	0.013
Post-operative complications, n (%)	1 (2.86)	2 (5.71)	1.000
Myasthenia crisis	0 (0)	0 (0)	
Pneumonia	0 (0)	1 (2.86)	
Pneumothorax	1 (2.86)	0 (0)	
Hemothorax	0 (0)	1 (2.86)	
Chylothorax	0 (0)	0 (0)	
Hospital stay (days), median (IQR)	5.00 (4.00, 7.00)	5.00 (3.00, 6.00)	0.141
30-day mortality	0 (0)	0 (0)	

Abbreviations: IQR interquartile range, RATS Robot-assisted thoracoscopic surgery, VATS Video-assisted thoracoscopic surgery

Notably, five patients in the VATS thymectomy group required conversion to open approaches, whereas none in the robotic group needed conversion, however, the difference was not statistically significant (0% vs. 14.29%, *P* = 0.054). Specifically, in one case, tumor invasion in the left pulmonary artery could not be excluded with thoracoscopy during surgery, and invasion of the phrenic nerve, pericardium, and left lung was also observed. After conversion to a sternotomy, the tumor was completely removed by resection with a segment of the left lung, part of the pericardium and the phrenic nerve. In two patients with suspected left innominate vein invasion, we tried to dissect the plane between the tumor and the innominate vein under thoracoscopy but failed. Hence, sternotomies were performed to avoid unexpected major bleeding. Due to the large diameter of the tumors that interfered with safe dissection, the other two patients were converted to open procedures.

The concomitant *en bloc* resection of adjacent structures with the thymus in the matched population with thymic neoplasms was comparable in both groups (11.43% vs. 5.71%; *P* = 0.673). Four patients required concomitant resection in the robotic group, including one patient with pulmonary wedge resection, two patients in whom the left innominate vein and partial pericardium were successfully resected and another patient with phrenic nerve, right lung and pericardium involvement who underwent complete resection through RATS as well. Two patients needed concomitant resection in the VATS group: one patient with pulmonary wedge resection and another patient with pericardium, lung and phrenic nerve involvement who required elective conversion. Additionally, no significant difference in the percentages of chest tube placements between the two groups was observed (97.14% vs. 91.43%; *P* = 0.614).

No myasthenia crises or 30-day mortality occurred after surgery in either group. No significant differences were identified in the duration of chest tube use (median: 3.00 vs. 3.00 days; *P* = 0.587), postoperative complications (2.86% vs. 5.71%; *P* = 1.000), or the duration of the postoperative hospital stay (median: 5.00 vs. 5.00 days; *P* = 0.141) between the two groups. However, compared with the VATS thymectomy group, patients in the RATS group had a larger pleural drainage volume after the operation (median: 575.00 vs. 400.00 ml; *P* = 0.013).

## Discussion

Although median sternotomy is still accepted as the standard treatment for thymic epithelial tumors, minimally invasive resections, mainly including VATS and RATS thymectomy, are becoming increasingly popular in clinical practice due to their great advantages in terms of a significantly reduced blood loss, less postoperative pain and rapid recovery. Larger thymic epithelial tumors (≥5.0 cm) or tumors involving major vascular infiltration with a substantially increased operative risk were once considered an absolute limiting factor for minimally invasive approaches [12, 13]. However, with the widespread adoption of the minimally invasive approach, several studies have reported that both VATS and RATS thymectomy are safe and effective approaches for large thymic epithelial tumors with comparable surgical and oncological results to open surgery [14, 15]. Furthermore, robotic surgery, which not only offers similar advantages but also overcomes the technical limitations of VATS thymectomy, has been regarded as a preferred approach in some studies [16]. However, due to the lack of comparison between the two approaches in the treatment of large thymic epithelial tumors, the choices of surgical approach have usually been based on the experience and preference of the surgeon and the patient’s willingness thus far. Therefore, we designed the present study to explore the more appropriate approach for larger thymic epithelial tumors. To our knowledge, this study is the first to compare the perioperative outcomes of VATS and RATS thymectomy for the treatment of large thymic epithelial tumors based on a propensity-matched analysis.

In the current study, we show that robotic thymectomy can achieve a similar complete surgical resection with free margins compared with a video-assisted procedure with comparable low postoperative complications and zero mortality. Although the robot set-up and docking time were included, a definite trend toward a reduction in the operative time was still easily observed in the robotic thymectomy group. Moreover, intra-operative blood loss volume shows more favorable result in the RATS group as well.

Consistent with previous studies, we defined a large thymic epithelial tumor as a tumor ≥ 5.0 cm in the current study [12, 14, 17]. The indicators of minimally invasive thymectomy (MIT) for a relatively large thymic epithelial tumor remain controversial. Most of studies indicate that tumors with a diameter < 5.0 cm are oncologically safe and MIT is technically feasible [18]. Nonetheless, several investigators have performed MIT to treat large thymomas and documented that radical resection can be performed safely and effectively [15, 19]. In the present study, radical resection was performed on all patients, and intraoperative injury and perioperative mortality were not observed. Therefore, from our perspective, tumor size is not an absolute contraindication for MIT. In contrast, MIT, particularly the RATS thymectomy, may provide excellent visualization for exposing the boundary between the tumor and healthy tissue, which is convenient for the resection of the invaded structures, even the great vessels. This result is similar to the study by Weng et al. which indicated that conversion to an open approach seems to mainly depend on tumor invasion to the great vessels regardless of the tumor size [14], and is consistent with our earlier study as well [20].

Although several studies have demonstrated that the VATS approach can achieve complete resection of large and advanced thymic epithelial tumors without conversion, the comparatively higher conversion rates in the current study might indicate that some technical limits of conventional thoracoscopy exist during the dissection of larger anterior mediastinal tumors. In the two-dimensional view of the operative field and under the physiologic hand tremor, the upper mediastinum with vulnerable large vessels and nerves becomes a delicate and difficult-to-dissect area [21]. Despite relatively proficient operation experience in thoracoscopic thymoma resection, surgeons can scarcely produce a fundamental change in these inherent limitations. By providing magnified 3D vision, flexible and finer instrument control and a stable operating system, the robotic surgical system overcomes several technical limitations of conventional thoracoscopy and is advantageous in dissection of larger thymic epithelial tumors requiring combined resection of adjacent structures. In fact, these advantages, which enable a better evaluation of the boundary between the tumor and healthy tissue and facilitate a more precise and low-risk dissection, have significantly increased the safety and expanded the indications of thymectomy for thymoma. Importantly, we preferred attempting RATS thymectomy first for almost every patient with large thymic epithelial tumors; however, an open approach would be decisively performed if complete resection could not be achieved during the operation, especially for patients with invasion of the aorta, pulmonary artery trunk, and atrium. Certainly, the initial robotic approach which helps surgeons dissect the thymoma and visualize areas of tumor invasion to facilitate subsequent open surgery should not be considered useless.

According to previous studies, the three main surgical approaches for thymoma include the unilateral thoracic cavity (left or right), bilateral thoracic cavity, and subxiphoid process. In the present study, the right-side thoracic approach was preferred in both groups, except for tumors located predominantly in the left mediastinum. Compared to the left-sided approach, the right-sided thoracic approach can be quite beneficial in avoiding interference from the heart and the aortic arch, and the left innominate vein may be identified and followed easily based on the visualization of the superior vena cava (SVC). Recently, several studies have documented the safety and feasibility of subxiphoid thymectomy for surgical resection of advanced thymic epithelial tumors, which allows the surgeon to visualize both phrenic nerves and reach high into the anterior mediastinum under direct vision [10, 22–24]. Since 2015, subxiphoid thymectomy has been gradually performed in our center, achieving comparable surgical and oncological results to transthoracic surgery. Sufficiently complete removal of the upper poles of the thymus in subxiphoid thymectomy has made it one of the routinely selected procedures for large thymic epithelial tumors at present.

The potential merit of RATS thymectomy in terms of enhanced recovery after surgery has already been noted in patients with early-stage thymic epithelial tumors [25]. Ye et al. analyzed the perioperative outcomes of patients with Masaoka stage I thymoma and found that robotic thymectomy was associated with a significantly shorter duration of chest drainage; and a shorter length of hospital stay [26]. Similar results were reported by Qian et al. in their study comparing three approaches for the treatment of early-stage thymomas [7]. However, our study showed that no difference in perioperative outcomes between patients who underwent RATS and those who underwent VATS. Even the total amount of postoperative drainage in the VATS group was less than that in the RATS group. We speculated that the primary cause of the discrepancy was a consequence of the relatively high proportion of patients requiring concomitant resection in the RATS group in the current study, implicating a potential need for additional manipulations and intraoperative dissections. In addition, omitting chest tube placement after surgery, which was more common in the VATS group, might be another contributing factor to this situation. Considering the higher rate of concomitant resection and relatively complex procedures performed in the RATS group, the similar postoperative parameters indicate that robotic thymectomy might have an advantage over VATS in postoperative rehabilitation, although further research is needed to confirm that.

In the present study, the retrospective nature, limited number of patients and single institution constituted major limitations. Especially, the decisions to perform excision with the use of VATS or the robotic system were not random and might lead to the selection biases. Although PSM was performed to improve comparability between the two groups, the results might be affected by the smaller sample sizes and potential biases. In addition, the follow-up was confined to the perioperative period, it was still inadequate to allow a definitive conclusion on oncologic outcome. Therefore, multicenter and randomized controlled studies comparing VATS and RATS thymectomy in patients with large thymic epithelial tumors are urgently warranted.

## Conclusion

The present study revealed that robotic-assisted thymectomy for large thymic epithelial tumors appears to be a safe and effective procedure. Due to advanced optics and precise instrument control, concomitant resections, particularly vascular resections can be easily achieved in patients with larger thymic epithelial tumors via a robotic approach, with equivalent perioperative results. Additional follow-up in future studies is required to evaluate the long-term oncological results of robotic thymectomy for thymic epithelial tumors.

## Data Availability

The datasets generated and analyzed during the present study are available from the corresponding author on reasonable request.

## Abbreviations

ASA: class Anesthesiologists physical status classification
CT: Computed tomography
ERAS: Enhanced recovery after surgery
IQRs: Interquartile ranges
MG: Myasthenia gravis
NSAIDs: Nonsteroidal anti-inflammatory drugs
PSM: Propensity score matching
RATS: Robot-assisted thoracoscopic surgery
SD: Standard deviations
SVC: Superior vena cava
VATS: Video-assisted thoracoscopic surgery
WHO: World Health Organization

## References

[1] Detterbeck FC, Zeeshan A (2013). Thymoma: current diagnosis and treatment. Chin Med J (Engl).

[2] Mao ZF, Mo XA, Qin C, Lai YR, Hackett ML (2012). Incidence of thymoma in myasthenia gravis: a systematic review. J Clin Neurol.

[3] National Comprehensive Cancer Network. NCCN Clinical Practice Guidelines in Oncology: Cancer-associated Venous Thromboembolic Disease; Version 2. 2022. https://www.nccn.org/professionals/physician_gls/pdf/vte.pdf (15 Nov 2022, date last accessed).

[4] Multidisciplinary Committee of Oncology, Chinese Physicians Association (2021). [Chinese guideline for clinical diagnosis and treatment of thymic epithelial tumors (2021 Edition)]. Zhonghua Zhong Liu Za Zhi.

[5] Friedant AJ, Handorf EA, Su S, Scott WJ (2016). Minimally invasive versus Open Thymectomy for Thymic malignancies: systematic review and Meta-analysis. J Thorac Oncol.

[6] Su KW, Luketich JD, Sarkaria IS (2022). Robotic-assisted minimally invasive thymectomy for myasthenia gravis with thymoma. JTCVS Tech.

[7] Qian L, Chen X, Huang J, Lin H, Mao F, Zhao X, Luo Q, Ding Z (2017). A comparison of three approaches for the treatment of early-stage thymomas: robot-assisted thoracic Surgery, video-assisted thoracic Surgery, and median sternotomy. J Thorac Dis.

[8] Shen C, Li J, Li J, Che G (2022). Robot-assisted thoracic Surgery versus video-assisted thoracic Surgery for treatment of patients with thymoma: a systematic review and meta-analysis. Thorac Cancer.

[9] Chen X, Ma Q, Wang X, Wang A, Huang D (2021). Subxiphoid and subcostal thoracoscopic surgical approach for thymectomy. Surg Endosc.

[10] Jiang L, Chen H, Hou Z, Qiu Y, Depypere L, Li J, He J (2022). Subxiphoid Versus Unilateral Video-assisted thoracoscopic Surgery thymectomy for Thymomas: a propensity score matching analysis. Ann Thorac Surg.

[11] Cui W, Huang D, Liang H, Peng G, Liu M, Li R, Xu X, He J (2021). Tubeless video-assisted thoracoscopic Surgery in mediastinal Tumor resection. Gland Surg.

[12] Kimura T, Inoue M, Kadota Y, Shiono H, Shintani Y, Nakagiri T, Funaki S, Sawabata N, Minami M, Okumura M (2013). The oncological feasibility and limitations of video-assisted thoracoscopic thymectomy for early-stage thymomas. Eur J Cardiothorac Surg.

[13] Casiraghi M, Galetta D, Borri A, Tessitore A, Romano R, Brambilla D, Maisonneuve P, Spaggiari L (2018). Robotic-assisted thymectomy for early-stage thymoma: a propensity-score matched analysis. J Robot Surg.

[14] Weng W, Li X, Meng S, Liu X, Peng P, Wang Z, Li J, Wang J (2020). Video-assisted thoracoscopic thymectomy is feasible for large thymomas: a propensity-matched comparison. Interact Cardiovasc Thorac Surg.

[15] Kneuertz PJ, Kamel MK, Stiles BM, Lee BE, Rahouma M, Nasar A, Altorki NK, Port JL (2017). Robotic thymectomy is feasible for large thymomas: a propensity-matched comparison. Ann Thorac Surg.

[16] Chiba Y, Miyajima M, Takase Y, Tsuruta K, Shindo Y, Nakamura Y, Ishii D, Sato T, Aoyagi M, Shiraishi T (2022). Robot-assisted and video-assisted thoracoscopic Surgery for thymoma: comparison of the perioperative outcomes using inverse probability of treatment weighting method. Gland Surg.

[17] Odaka M, Tsukamoto Y, Shibasaki T, Katou D, Mori S, Asano H, Yamashita M, Morikawa T (2017). Thoracoscopic thymectomy is a feasible and less invasive alternative for the surgical treatment of large thymomas. Interact Cardiovasc Thorac Surg.

[18] Marulli G, Rea F, Melfi F, Schmid TA, Ismail M, Fanucchi O, Augustin F, Swierzy M, Di Chiara F, Mussi A (2012). Robot-aided thoracoscopic thymectomy for early-stage thymoma: a multicenter European study. J Thorac Cardiovasc Surg.

[19] Agatsuma H, Yoshida K, Yoshino I, Okumura M, Higashiyama M, Suzuki K, Tsuchida M, Usuda J, Niwa H (2017). Video-assisted thoracic Surgery Thymectomy Versus Sternotomy Thymectomy in patients with Thymoma. Ann Thorac Surg.

[20] Jiang B, Tan QY, Deng B, Mei LY, Lin YD, Zhu LF (2023). Robot-assisted thymectomy in large anterior mediastinal tumors: a comparative study with video-assisted thymectomy and open Surgery. Thorac Cancer.

[21] Marulli G, Maessen J, Melfi F, Schmid TA, Keijzers M, Fanucchi O, Augustin F, Comacchio GM, Mussi A, Hochstenbag M (2016). Multi-institutional European experience of robotic thymectomy for thymoma. Ann Cardiothorac Surg.

[22] Suda T (2017). Subxiphoid thymectomy: single-port, dual-port, and robot-assisted. J Vis Surg.

[23] Na KJ, Kang CH (2020). Robotic thymectomy for advanced thymic epithelial Tumor: indications and technical aspects. J Thorac Dis.

[24] Kang CH, Na KJ, Song JW, Bae SY, Park S, Park IK, Kim YT (2020). The robotic thymectomy via the subxiphoid approach: technique and early outcomes. Eur J Cardiothorac Surg.

[25] Şehitogullari A, Nasır A, Anbar R, Erdem K, Bilgin C (2020). Comparison of perioperative outcomes of videothoracoscopy and robotic surgical techniques in thymoma. Asian J Surg.

[26] Ye B, Tantai JC, Li W, Ge XX, Feng J, Cheng M, Zhao H (2013). Video-assisted thoracoscopic Surgery versus robotic-assisted thoracoscopic Surgery in the surgical treatment of Masaoka stage I thymoma. World J Surg Oncol.

